# Integrative analysis of exosomal microRNA-149-5p in lung adenocarcinoma

**DOI:** 10.18632/aging.202596

**Published:** 2021-02-26

**Authors:** Wen Tian, He Yang, Baosen Zhou

**Affiliations:** 1 Department of Clinical Epidemiology, First Affiliated Hospital, China Medical University, Shenyang, China; 2 Department of Epidemiology, School of Public Health, China Medical University, Shenyang, China

**Keywords:** exosomal miR-149-5p, WGCNA, AMOTL2, growth, apoptosis

## Abstract

Exosomes play important roles in the regulation of various processes in the tumor microenvironment. In this study, we explored the mechanisms of exosomal miR-149-5p in the pathogenesis of lung adenocarcinoma. Raw data were downloaded and normalized using the R package. Significantly expressed exosomal miRNAs were subjected to co-expression network analysis. The proliferation and apoptotic abilities of tumor cells were assessed by the proliferation and apoptosis assays. Univariate and multivariate analyses were performed to identify the independent risk factors of exosomal miR-149-5p and AMOTL2. Results showed that exosomal miR-149-5p was enriched in peripheral serum and tumor cells. The upregulation of exosomal miR-149-5p promoted the growth of tumor cells and inhibited apoptosis of tumor cells. Notably, AMOTL2, the target gene of exosomal miR-149-5p, was significantly downregulated in lung adenocarcinoma and may be considered as an independent risk factor of poor survival. In lung adenocarcinoma cells, AMOTL2 downregulation reversed the promoting effect of miR-149-5p on A549 cells growth and the inhibition effect of miR-149-5p on A549 cells apoptosis. Collectively, these results provide specific insights for further mechanistic studies on lung adenocarcinoma.

## INTRODUCTION

Lung cancer is the leading cause of cancer-related deaths in the world [1]. In China, an estimated 733,300 new lung cancer cases and 610,200 lung cancer deaths were recorded in 2015 [2]. Lung adenocarcinoma is a complicated subtype of lung cancer [3]. Numerous studies have explored the molecular mechanisms of this cancer [4–6]. In our study, we investigated its pathomechanism to provide reliable diagnostic and therapeutic biomarkers in lung adenocarcinoma.

AMOTL2 is a member of Amot family [7] which could interfere cells polarity to promote the migration ability [8]. The results show that AMOTL2 plays a vital role in migration of endothelial cells [9]. AMOTL2 functions as an oncogene or anti-oncogene in different human tumors. For instance, AMTOL2 induces the angiogenesis of pancreatic cancer through UCA1/miR-96-5p/AMOTL2 axis [10]. mTORC2/AMOTL2/YAP axis promotes the proliferation and invasiveness of glioblastoma cells [11]. However, in breast cancer, high expression level of AMOTL2 was significantly negative correlated with clinical grade [12]. In non-small cell lung cancer, AMOTL2 functioned as negative regulator of tumor cells proliferation via AMOTL2/PPP2R2A/JUN axis [13]. Thus, it is urgent to explore the detailed biological mechanism of AMOTL2 in lung adenocarcinoma.

Exosomes are endosomes-derived vesicles with the sizes in the range of 40-150nm [14]. They transport RNAs and proteins to recipient cells [15], and regulate tumor microenvironment [16–18]. In breast cancer, it was found that MDA-MB-231 cells released exosomes containing miR-10b. The exosomal miR-10b suppressed the invasion ability of HMLE cells [19]. Elsewhere, exosomal miR-382-5p released from oral squamous cell carcinoma cells stimulated tumor cells migration and invasion ability [20]. In non-small cell lung cancer, BMMSC-derived exosomes transferred miR-193a to induce cisplatin resistance via suppressing LRRC1 [21]. In previous studies, miR-149-5p was found to be highly expressed in lung adenocarcinoma cells and influenced tumor cells metastasis [22, 23]. However, the roles of exosomal miR-149-5p in lung adenocarcinoma remain unclear.

In this study, we constructed a WGCNA co-expression network to identify the key exosomal miRNA. Moreover, we explored that exosomal miR-149-5p accelerated growth and suppressed apoptosis of tumor cells via inhibiting AMOTL2.

## RESULTS

### Differently expressed exosomal miRNAs in GSE111803

**Figure 1:**
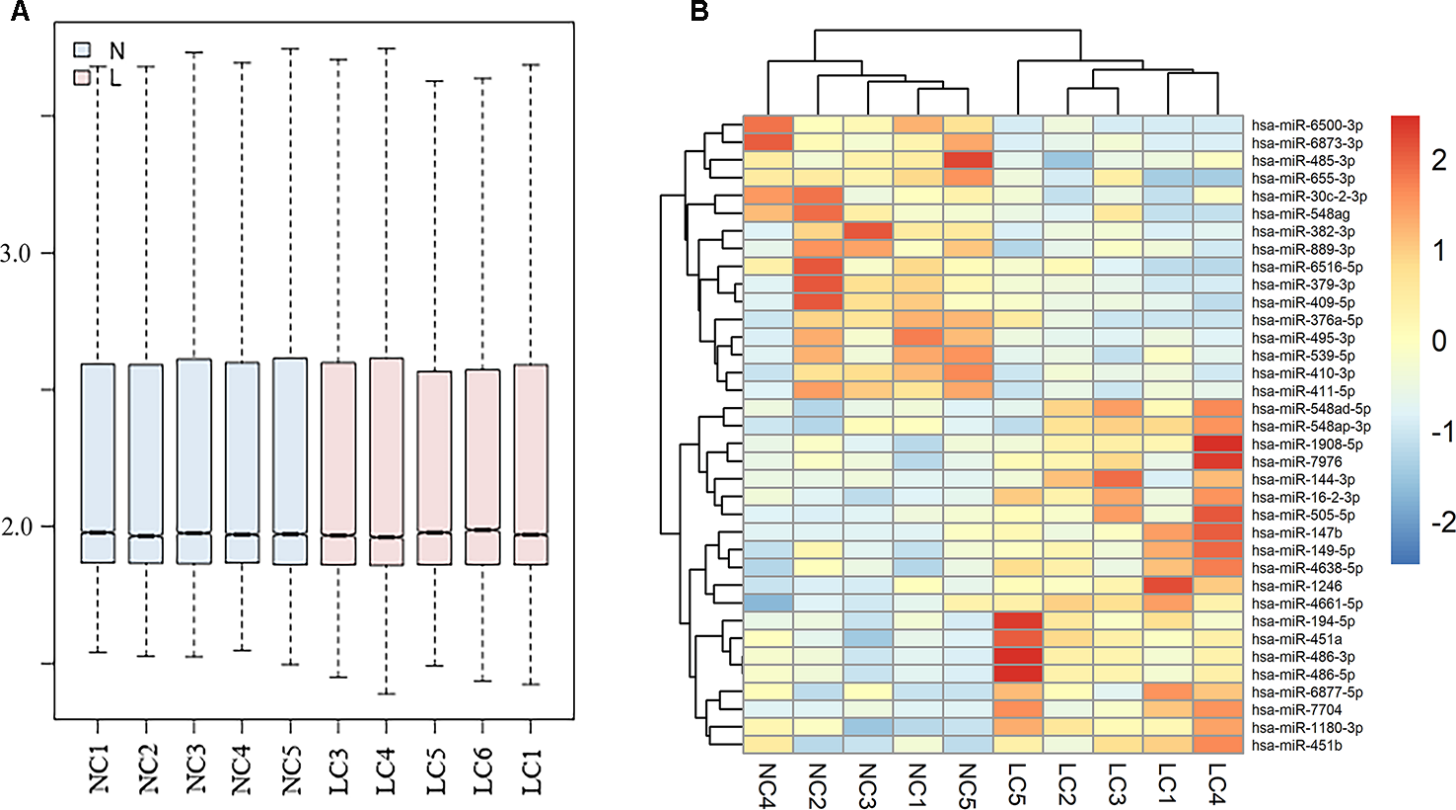
**Differently expressed exosomal miRNAs in GSE111803.** (**A**) Normalization of raw data in GSE111803. (**B**) The heat map of differently expressed exosomal miRNAs in GSE111803. (NC: negative control; LC: lung adenocarcinoma).

Normalized data for each group in GSE111803 was showed in Figure 1A. The result plotted the similar expression distribution in each group. According to the criterion of adjusted p<0.05 and |log2FC|>2, a total of 559 miRNAs were identified. Among them, 36 significantly expressed exosomal miRNAs were displayed in the heat map (Figure 1B).

### WGCNA co-expression network of exosomal miRNAs

**Figure 2:**
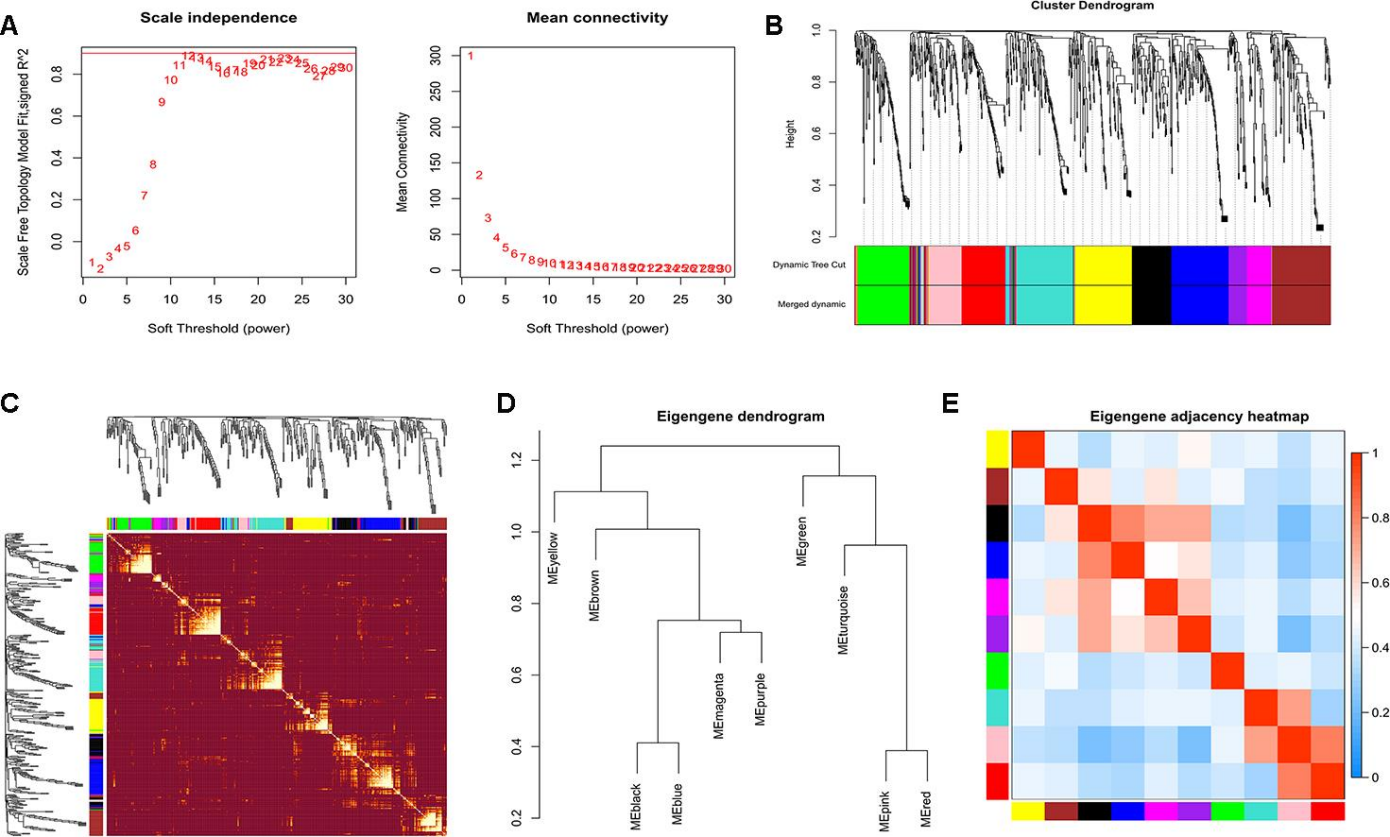
**Construction of WGCNA.** (**A**) Analyses of scale-free fit index and mean connectivity for various soft-thresholding powers. (**B**) Clustering dendrograms of exosomal miRNAs based on a dissimilarity measure. (**C**) The hierarchical clustering dendrograms correspond to each module. (**D**) Hierarchical clustering of each exosomal miRNAs module. (**E**) The heatmap plot of the adjacencies in the exosomal miRNAs modules.

The power of β=12 was chosen as the soft thresholding power value when the scale free R^2^=0.95 (Figure 2A). A total of 10 modules were identified (Figure 2B). Each module had different numbers of exosomal miRNAs (Table 1). The modules were highly independent of each other (Figure 2C). In addition, to explore co-expression similarity of each module, we calculated the eigengenes based on their correlation (Figure 2D). These ten modules yielded two main clusters; one contained six modules, while the other contained four modules. The heatmap plot of the adjacencies also supported this result (Figure 2E). Furthermore, we explored the relation between ten modules and lung adenocarcinoma (Table 2). The results showed that the turquoise module had the strongest connection (*P*<0.001) with NSCLC (hsa05223). In turquoise module, we found that the function of exosomal miR-149-5p in lung adenocarcinoma remained unclear. Thus, we investigated the role of exosomal miR-149-5p for the following experiment.

**Table 1:** Numbers of exosomal miRNAs in each module.

**Color**	**Number**	**Color**	**Number**
Black	49	Pink	43
Blue	72	Purple	23
Brown	71	Red	61
Green	65	Turquoise	75
Magenta	31	Yellow	69

**Table 2:** Pathways of exosomal miRNAs in each module.

**Color**	**Pathways**	***P* value**	**Color**	**Pathways**	***P* value**
Black	Pathways in cancer (hsa05200)	1.419568e-12	Blue	PI3K-Akt signaling pathway (hsa04151)	1.102411e-10
	Bladder cancer (hsa05219)	1.95996e-11		Pathways in cancer (hsa05200)	1.922688e-10
	Pancreatic cancer (hsa05212)	7.393284e-10		ErbB signaling pathway(hsa04012)	1.089081e-09
	PI3K-Akt signaling pathway (hsa04151)	1.277962e-09		MAPK signaling pathway (hsa04010)	6.162801e-09
	Cell cycle (hsa04110)	1.669804e-07		**NSCLC (hsa05223)**	**1.646353e-07**
Brown	ErbB signaling pathway (hsa04012)	1.015039e-24	Green	MAPK signaling pathway (hsa04010)	2.040638e-19
	Neurotrophin signaling pathway (hsa04722)	1.068046e-24		Wnt signaling pathway (hsa04310)	6.65386e-16
	PI3K-Akt signaling pathway (hsa04151)	4.017153e-19		ErbB signaling pathway (hsa04012)	1.039351e-11
	Colorectal cancer (hsa05210)	1.259041e-18		Transcriptional misregulation in cancer (hsa05202)	3.932956e-10
	Wnt signaling pathway (hsa04310)	1.169783e-17		Pathways in cancer (hsa05200)	1.366175e-09
Magenta	Transcriptional misregulation in cancer (hsa05202)	3.953364e-17	Pink	Glutamatergic synapse (hsa04724)	4.911598e-11
	Focal adhesion (hsa04510)	1.703439e-16		Wnt signaling pathway (hsa04310)	1.523735e-10
	ErbB signaling pathway (hsa04012)	8.688603e-15		Neurotrophin signaling pathway (hsa04722)	6.150503e-09
	T cell receptor signaling pathway (hsa04660)	1.116865e-14		MAPK signaling pathway (hsa04010) 2.41413e-08	2.41413e-08
	PI3K-Akt signaling pathway (hsa04151)	3.05292e-12		ErbB signaling pathway (hsa04012)	2.639617e-07
Purple	Ubiquitin mediated proteolysis (hsa04120)	1.233083e-12	**Red**	mTOR signaling pathway (hsa04150)	2.480427e-18
	Prion diseases (hsa05020)	8.092908e-12		Prostate cancer (hsa05215)	1.006374e-17
	Colorectal cancer (hsa05210)	8.092908e-12		Pathways in cancer (hsa05200)	1.051852e-16
	Focal adhesion (hsa04510)	1.271225e-11		PI3K-Akt signaling pathway (hsa04151)	1.836449e-16
	TGF-beta signaling pathway (hsa04350)	3.088237e-09		ErbB signaling pathway (hsa04012)	3.106143e-15
Turquoise	mTOR signaling pathway (hsa04150)	2.480427e-18	**Yellow**	mTOR signaling pathway (hsa04150)	2.480427e-18
	**NSCLC (hsa05223)**	1.006374e-17		Prostate cancer (hsa05215)	1.006374e-17
	Pathways in cancer (hsa05200)	1.051852e-16		Pathways in cancer (hsa05200)	1.051852e-16
	PI3K-Akt signaling pathway (hsa04151)	1.836449e-16		PI3K-Akt signaling pathway (hsa04151)	1.836449e-16
	ErbB signaling pathway (hsa04012)	3.106143e-15		**NSCLC (hsa05223)**	6.148222e-15

### Exosomal miR-149-5p promoted lung adenocarcinoma cells proliferation

**Figure 3:**
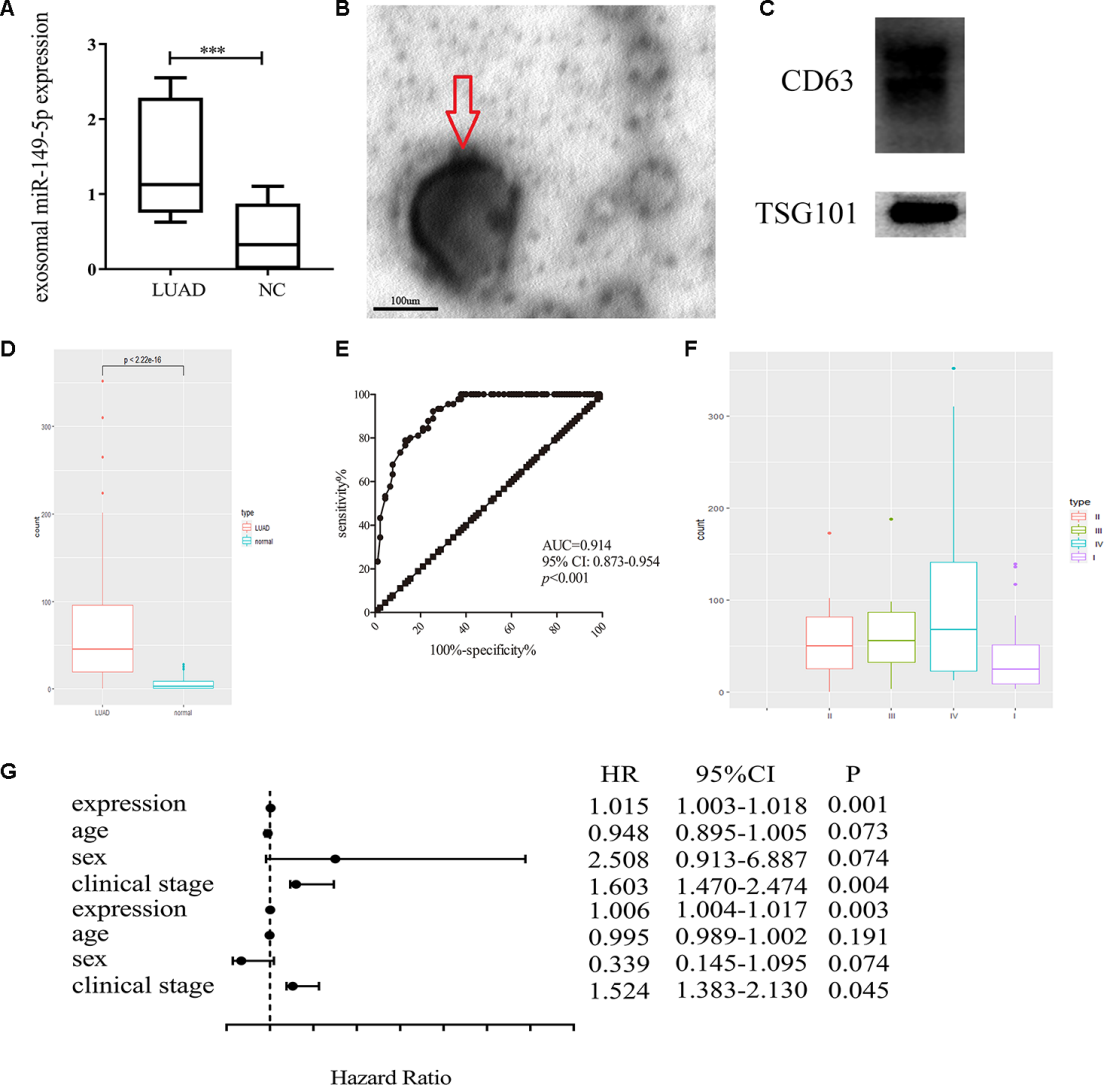
**Exosomal miR-149-5p was considered as a biomarker of lung adenocarcinoma.** (**A**) Upregulation of exosomal miR-149-5p in lung adenocarcinoma compared to healthy controls in GSE111803. (**B**) The transmission electron micrograph of A549 cells exosomes. (**C**) Western blot analyses for exosomal markers, CD63 and TSG101. (**D**) Upregulation of exosomal miR-149-5p in lung adenocarcinoma patients. (**E**) ROC curve analysis of exosomal miR-149-5p in lung adenocarcinoma. (**F**) The expression level of exosomal miR-149-5p in different clinical stage. (**G**) The univariate regression and multivariate regression analysis of exosomal miR-149-5p. LUAD: lung adenocarcinoma; AUC: area under curve; HR: hazard ratio; CI: Confidence Interval.

Firstly, we found the upregulation of exosomal miR-149-5p in peripheral serum of lung adenocarcinoma patients in GSE111803 (Figure 3A). Then we isolated exosomes from conditioned media and identified the cup-shaped structure and size by electron microscopy (Figure 3B). In addition, we detected the known exosome biomarkers, CD63 and TSG101 (Figure 3C). The results verified that the isolated particles were exosomes.

Additionally, the up-regulation of exosomal miR-149-5p was significantly observed in patients with lung adenocarcinoma (Figure 3D). The ROC curve analysis showed that exosomal miR-149-5p had better diagnostic value in lung adenocarcinoma (Figure 3E: AUC=0.914, p<0.001). Figure 3F revealed that the expression level of exosomal miR-149-5p was higher in advanced clinical stage. The result of univariate and multivariate analyses showed that exosomal miR-149-5p could be considered as an independent risk factor in lung adenocarcinoma (Figure 3G).

**Figure 4:**
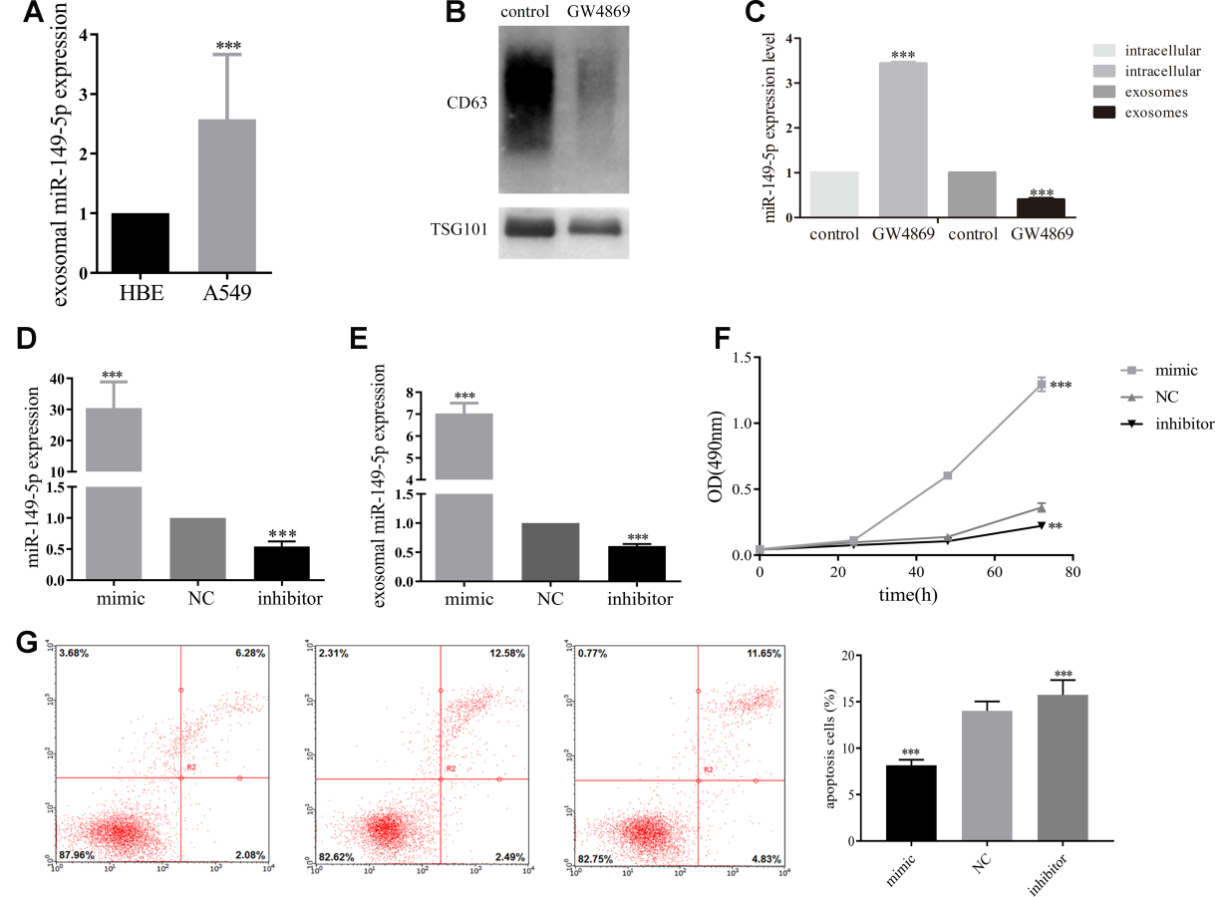
**Exosomal miR-149-5p promoted lung adenocarcinoma cells proliferation.** (**A**) Upregulation of exosomal miR-149-5p in A549 cells compared to HBE cells. (**B**) The effect of GW4869 in A549 cells. (**C**) The expression level of exosomal miR-149-5p in cells and exosomes with the treatment of GW4869. (**D**) The expression of miR-149-5p in A549 cells with different treatments. (**E**) The expression of exosomal miR-149-5p in A549 cells with different treatments. (**F**) The growth abilities of A549 cells with different treatments. (**G**) The apoptotic rates of A549 cells with different treatments. **p<0.01, ***p<0.001.

Furthermore, to explore the effect of exosomal miR-149-5p on lung adenocarcinoma tumor cells, we firstly profiled the expression secreted from HBE and A549 cells. Exosomal miR-149-5p was significantly up-regulated in A549 cells compared to HBE cells (Figure 4A). Firstly, we utilized GW4869 to inhibit the secretion of exosomes from A549 cells (Figure 4B). Then we found that the expression level of miR-149-5p was significantly higher in GW4869 group than control group (Figure 4C). Conversely, the expression level of miR-149-5p in exosomes was significantly lower in GW4869 group than control group.

Then we transfected miR-149-5p-mimic, miR-149-5p-inhibitor and NC into A549 cells. We found a 30-fold increase of miR-149-5p in cells transfected with miR-149-5p-mimic compared to NC (Figure 4D). A 3-fold decrease of miR-149-5p in cells transfected with miR-149-5p-inhibitor was detected. Next, we measured the expression of exosomal miR-149-5p in different groups. The RT-PCR assays results revealed the overexpression of exosomal miR-149-5p in miR-149-5p-mimic cells by over 7-fold and low-expression in miR-149-5p-inhibitor cells by over 2-fold compared to the NC (Figure 4E).

As shown in Figure 4F, MTS assays showed that up-regulation of exosomal miR-149-5p promoted the growth of A549 cells and this function was blocked by exosomal miR-149-5p-inhibitor.

### Up-regulation of exosomal miR-149-5p suppressed lung adenocarcinoma cells apoptosis

As shown in Figure 4G, the apoptotic rates of A549 cells transfected with miR-149-5p-inhibitor was significantly higher than cells transfected with NC and miR-149-5p-mimic.

### Integrative analyses of target genes of exosomal miR-149-5p

**Figure 5:**
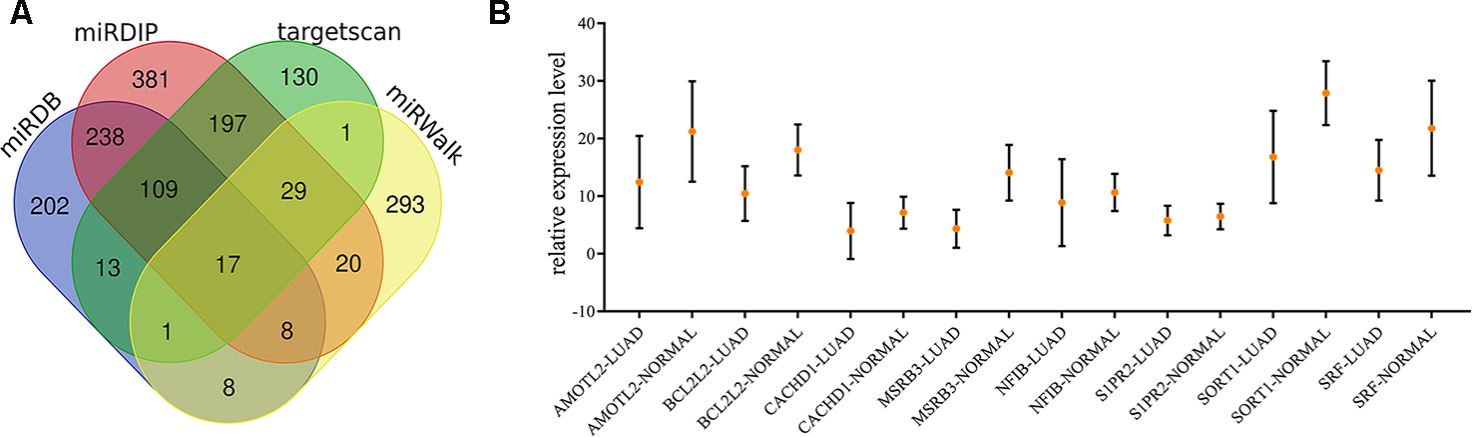
**Identification of target genes of exosomal miR-149-5p.** (**A**) Venn map of common target genes. (**B**) The expression profile of 8 target genes in TCGA-LUAD cohort.

**Figure 6:**
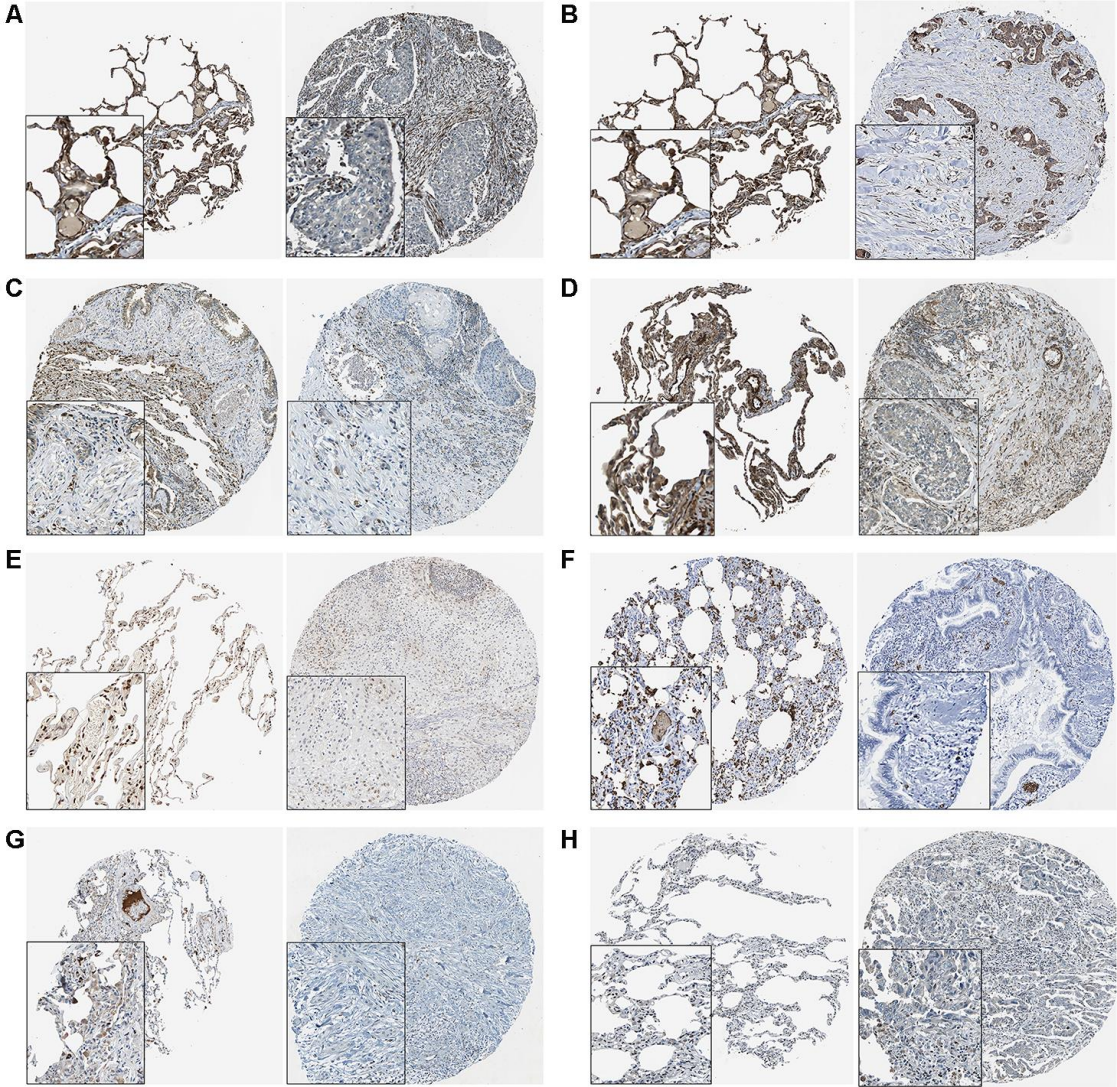
**The THPA results of 8 target genes in normal and tumor tissues.** (**A**) AMOTL2. (**B**) BCL2L2. (**C**) CACDH1. (**D**) MSRB3. (**E**) NFIB. (**F**) S1PR2. (**G**) SORT1. (**H**) SRF.

We summarized the target genes from Targetscan, miRWalk, miRDB and miRDIP bioinformatic websites and a total of 17 common genes were identified (Figure 5A). We observed that 8 target genes, AMOTL2, BCL2L2, CACHD1, MSRB3, NFIB, S1PR2, SORT1 and SRF, were significantly down-regulated in lung adenocarcinoma (Figure 5B). Results from THPA data also revealed the decreased expression of those genes (Figure 6A–6H) in lung adenocarcinoma tissues.

**Figure 7:**
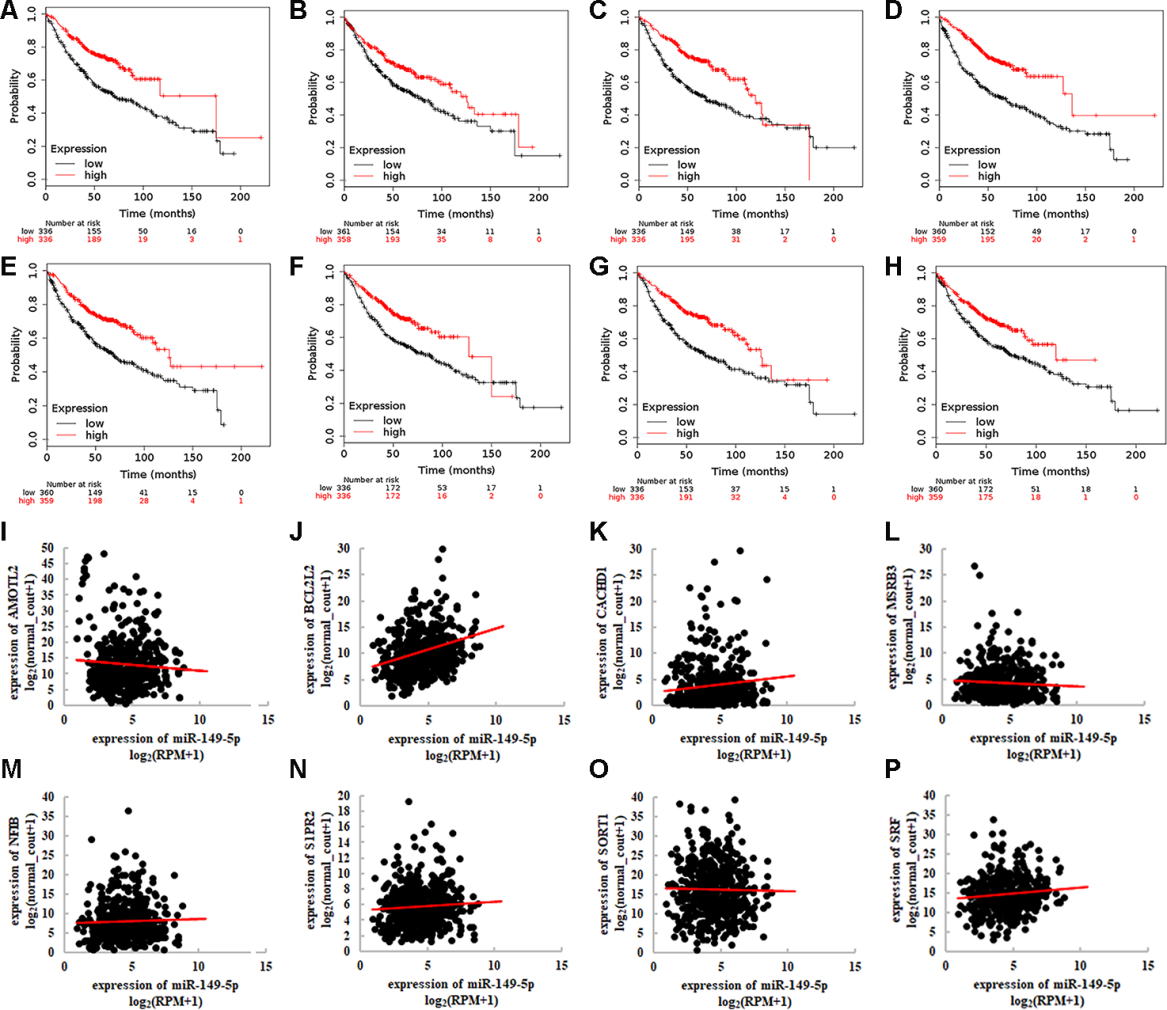
**Integrative analyses of 8 target genes of exosomal miR-149-5p**. Survival analysis of 8 target genes in TCGA-LUAD cohort (**A**) AMOTL2. (**B**) BCL2L2. (**C**) CACDH1. (**D**) MSRB3. (**E**) NFIB. (**F**) S1PR2. (**G**) SORT1. (**H**) SRF. Correlation between 8 target genes and miR-149-5p expression in TCGA-LUAD cohort (**I**) AMOTL2. (**J**) BCL2L2. (**K**) CACDH1. (**L**) MSRB3. (**M**) NFIB. (**N**) S1PR2. (**O**) SORT1. (**P**) SRF. RPM: reads of exon model per million mapped reads.

Then we investigated the association between 8 target genes and overall survival of lung adenocarcinoma (Figure 7A–7H). The Hazard Ratios were 0.520 (95%CI: 0.400-0.670; *P*=2.700e-07), 0.650 (95%CI: 0.520-0.830; *P*=3.300e-03), 0.520 (95%CI: 0.400-0.670; *P*=1.700e-07), 0.440 (95%CI: 0.350-0.570; *P*=4.900e-11), 0.530 (95%CI: 0.420-0.680; *P*=2e-07), 0.570 (95%CI: 0.440-0.740; *P*=1.500e-05), 0.520 (95%CI: 0.400-0.660; *P*=1.300e-07), 0.620 (95%CI: 0.480-0.790; *P*=8.900e-05), respectively. We explored the relationship between 8 target genes and miR-149-5p (Figure 7I–7P). The results revealed the significant association between AMOTL2 and miR-149-5p (Figure 7I: r=-0.354, P<0.001).

### Exosomal miR-149-5p mediated the proliferation and apoptosis abilities of tumor cells by targeting AMOTL2

**Figure 8:**
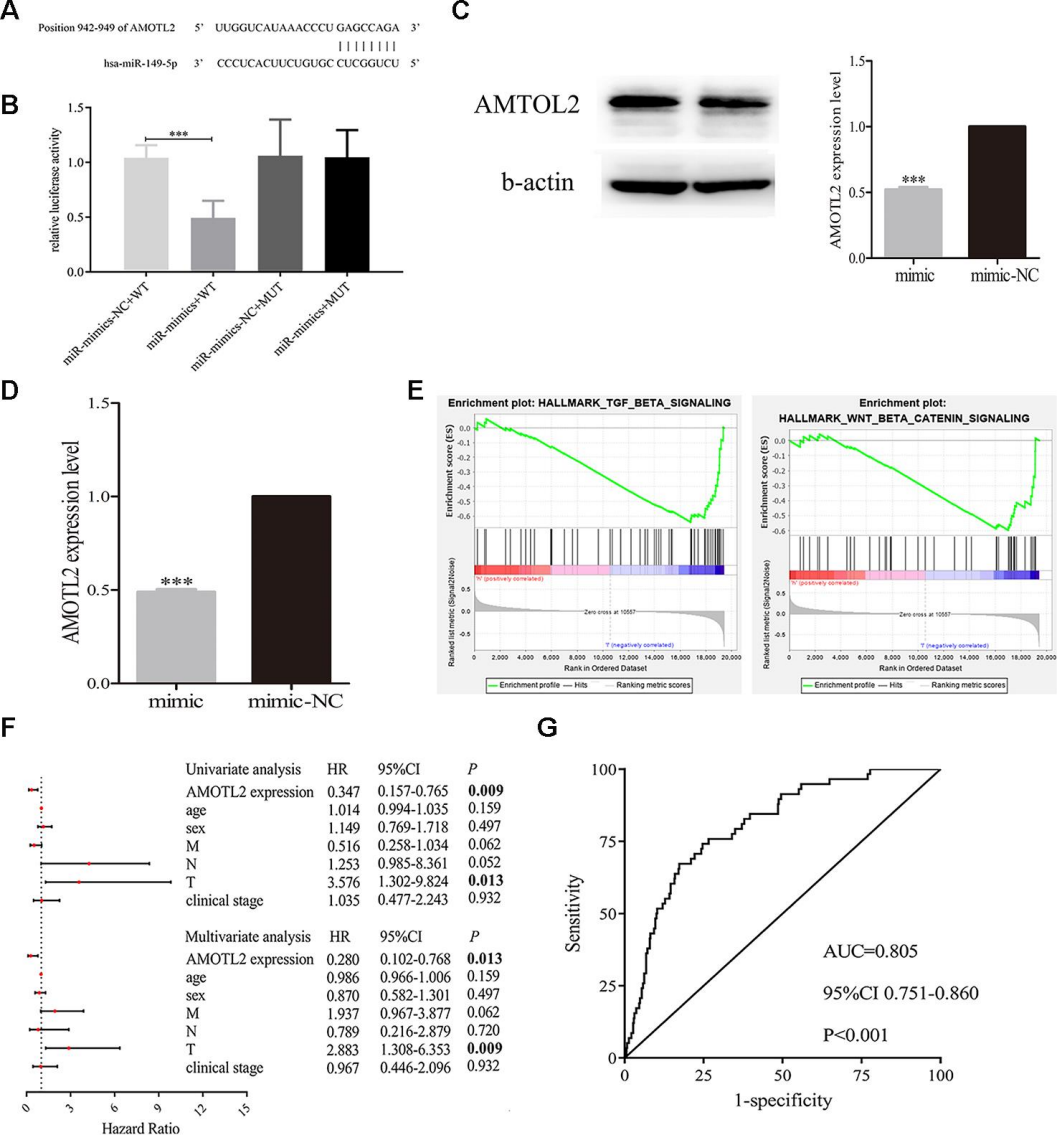
**Exosomal miR-149-5p regulated the proliferation and apoptotic rates of tumor cells by targeting AMOTL2**. (**A**) The binding site of miR-149-5p and AMOTL2. (**B**) Dual luciferase reporter gene assay of exosomal miR-149-5p and AMOTL2. (**C**) The AMOTL2 protein expression in cells transfected with miR-149-5p mimic and mimic NC. (**D**) The AMOTL2 mRNA expression in cells transfected with miR-149-5p mimic and mimic NC. (**E**) GSEA results of AMOTL2 in TCGA-LUAD cohort. (**F**) Univariate and multivariate analyses of AMOTL2 in TCGA-LUAD cohort. (**G**) ROC curve analysis of AMOTL2 in TCGA-LUAD cohort. ***p<0.001 (WT: wild type; MUT: mutant type).

Firstly, we predicted the binding site of miR-149-5p and AMOTL2 from bioinformatics online (Figure 8A). Then we performed dual luciferase reporter assays in A549 cells. The results showed that luciferase reporter activity was inhibited in cells co-transfected with miR-149-5p mimic and wild-type AMOTL2 3’UTR compared with cells co-transfected with miR-149-5p NC and wild-type AMOTL2 3’UTR (Figure 8B). However, there were no significant differences in the dual luciferase of cells transfected with mut-type. Additionally, we found that the expression levels of AMOTL2 protein and mRNA were significantly lower in miR-149-5p mimic group than miR-149-5p mimic NC group (Figure 8C, 8D). Thus, these results indicated that miR-149-5p could directly sponge with AMTOL2.

GSEA results indicated that AMOTL2 could regulate the development of lung adenocarcinoma via TGF-β signaling pathway and Wnt/β signaling pathway (Figure 8E). The result of univariate and multivariate analyses showed that AMOTL2 could be considered as an independent risk factor in lung adenocarcinoma (Figure 8F). The ROC curve analysis result revealed the better prognostic ability of AMOTL2 in TCGA-LUAD cohort (Figure 8G: AUC=0.805, *P*<0.001).

**Figure 9:**
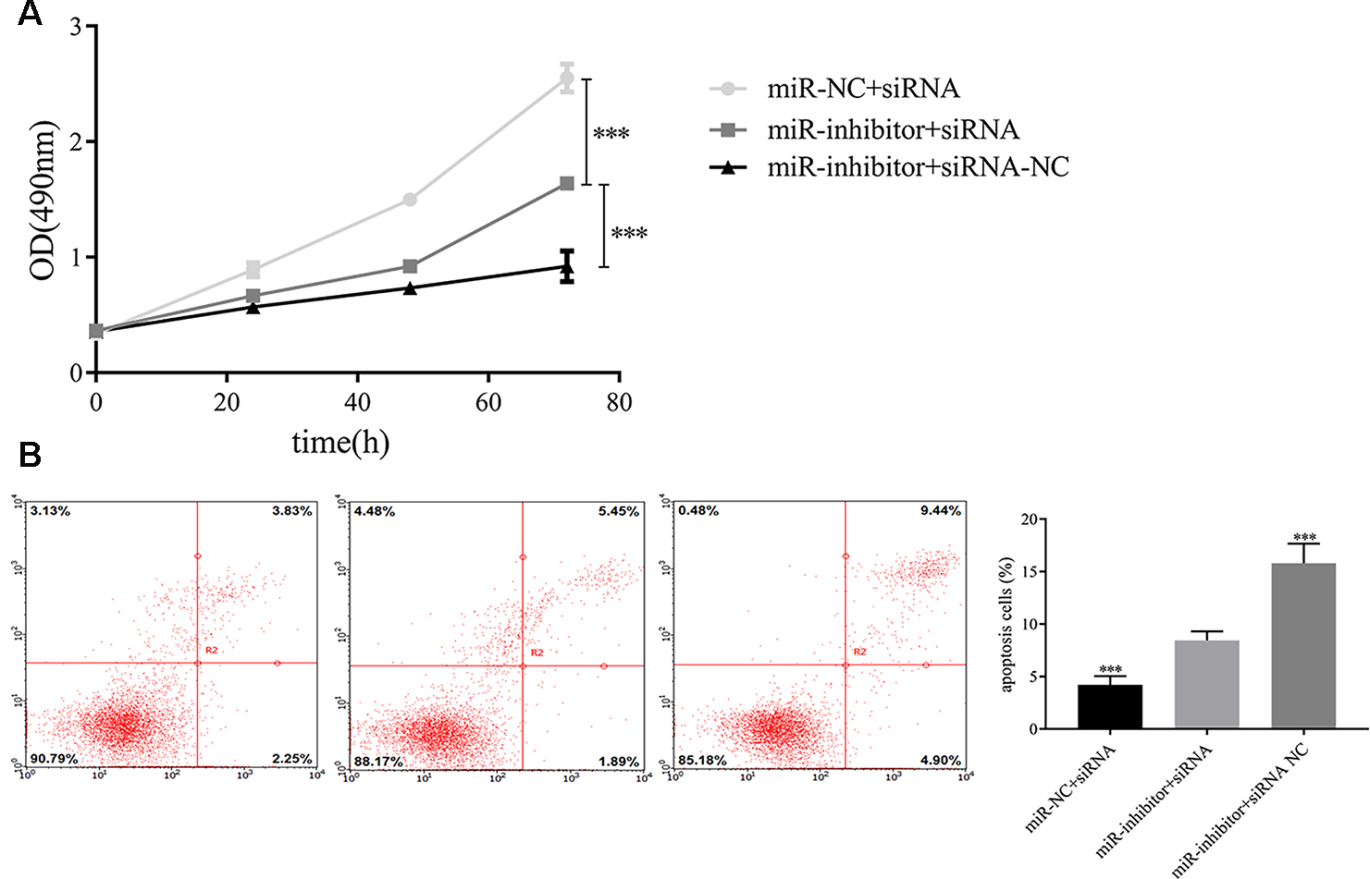
**Exosomal miR-149-5p regulated the proliferation and apoptotic rates of tumor cells by targeting AMOTL2.** (**A**) Exosomal miR-149-5p promoted the proliferation of tumor cells by targeting AMOTL2. (**B**) Exosomal miR-149-5p inhibited the apoptotic rates of tumor cells by targeting AMOTL2.***p<0.001.

Additionally, we detected whether exosomal miR-149-5p regulated the growth and apoptosis of tumor cells by inhibiting AMOTL2. We transfected AMOTL2-siRNA or AMOTL2-NC and miR-149-5p inhibitor or miR-149-5p NC into A549 cells. We found that the downregulation of AMOTL2 reversed the impacts of miR-149-5p downregulation on proliferation (Figure 9A). The apoptotic assay showed that the promotive effect of miR-149-5p inhibitor on the apoptotic abilities of tumor cells could be rescued by the inhibition of AMOTL2 (Figure 9B).

## DISCUSSION

Exosomal microRNAs play vital roles in the occurrence and development of tumors [15, 24, 25]. Exosomal miR-155 elevated growth rates of tumor cells in gastric carcinoma via c-MYB/VEGF axis [26]. Up-regulation of exosomal miR-146b-5p and miR-222-3p augmented migration and invasion abilities of papillary thyroid carcinomas [27]. Additionally, it was found that BMMSC-derived exosomal miR-144 suppressed growth of tumor cells and colony formation in NSCLC by targeting CCNE1 and CCNE2 [28]. In colorectal cancer, overexpression of exosomal miR-106b-3p was significantly associated with poor prognosis [29].

In our study, we firstly analyzed the raw data of GSE111803 and found that exosmal miR-149-5p was highly expressed in peripheral serum of lung adenocarcinoma patients. Thus, we hypothesized that exosomal miR-149-5p may be function as an oncogene in lung adenocarcinoma. A total of 90 couples was used to further verify the higher expression of exosomal miR-149-5p in patients with lung adenocarcinoma. Additionally, ROC curve analysis result revealed the better diagnostic value of exosomal miR-149-5p in lung adenocarcinoma. Furthermore, the expression level of exosomal miR-149-5p was positively related with clinical stage. The results of univariate and multivariate regression analyses indicated that the expression of exosomal miR-149-5p could be considered as an independent prognostic factor in patients with lung adenocarcinoma. These results validate the hypothesis that exosomal miR-149-5p may act as an oncogene in lung adenocarcinoma.

RT-PCR assay results confirmed that exosomal miR-149-5p was highly expressed in A549 cells. We used GW4869 to prove that exosomes from A549 cells contained miR-149-5p. After the treatment of GW4869, A549 cells significantly secreted less exosomes. Following the expression of miR-149-5p in exosomes was significantly down-regulated. On the contrary, the expression of miR-149-5p in intracellular was up-regulated. Above all, exosomes from A549 cells contained miR-149-5p which could transfer to HBE cells to regulate the microenvironment. MTS assay demonstrated that exosomal miR-149-5p stimulated proliferation of tumor cells. In contrast, it inhibited apoptosis of A549 cells. These findings point to a potential role of exosomal miR-149-5p as an alternative biomarker for lung adenocarcinoma.

To identify the downstream targets of exosomal miR-149-5p, bioinformatics tools were used to predict target genes. Among them, 8 genes were aberrantly expressed in lung adenocarcinoma. The immunohistochemical results also proved the similar tendency. The survival analyses of those genes revealed the protective factors in lung adenocarcinoma. Further, the results of expression analyses in TCGA-LUAD revealed that miR-149-5p was a negative regulator of AMOTL2, indicating that exosomal miR-149-5p regulated metastasis of tumor cells by targeting AMOTL2 gene.

The AMOT gene family members function as tumor suppressors in several human cancers. AMOT was significantly downregulated in lung cancer tissues and tumor cells [30]. Particularly, AMOTL2 played an important regulatory role in tumor microenvironment. In breast cancer, AMOTL2 increased LATS kinase activity leading to the suppression of metastasis of tumor cells [31]. Besides, when downregulated, AMOTL2 enhanced tumor cells growth [32], migration and angiogenesis [33] via Hippo pathway in hepatocellular carcinoma. However, the effect of AMOTL2 on lung adenocarcinoma is incompletely understood.

In our study, AMOTL2 was found to be tumor suppressor gene in TCGA-LUAD cohort. Univariate and multivariate analyses results showed the low expressions of AMOTL2 was an independent risk factor for predicting overall survival in lung adenocarcinoma. Moreover, GSEA results indicated that AMOTL2 could regulate the progression of lung adenocarcinoma via TGF-β signaling pathway and Wnt/β signaling pathway. Numerous studies had demonstrated the biological functions of TGF-β signaling pathway [34–37] and Wnt/β signaling pathway [38–41] in lung adenocarcinoma, indicating that AMOTL2 played a vital role in the occurrence and development of lung adenocarcinoma. The dual luciferase reporter assay indicated that miR-149-5p regulated the expression of AMOTL2. The complementation assays demonstrated that AMOTL2 reversed the effects of miR-149-5p on growth and apoptosis. Consistently, exosomal miR-149-5p promoted growth and inhibited apoptosis of tumor cells via targeting AMOTL2 gene.

In summary, we analyzed GSE111803 raw data and identified genes for construction of a WGCNA co-expression network. Our results demonstrated that exosomal miR-149-5p activated the growth and reduced apoptotic rate of tumor cells in lung adenocarcinoma via targeting AMOTL2. Our study suggests that exosomal miR-149-5p may be a reliable tumor microenvironment biomarker in lung adenocarcinoma.

## MATERIALS AND METHODS

### Raw data collection

Raw data were downloaded from GEO and normalized using the R package. GSE111803 contained 5 lung adenocarcinoma patients and 5 healthy controls. Adjusted p<0.05 and |log_2_FC|>1 were set as the criterion for differently expressed exosomal miRNAs. Raw count data and clinical characteristics of TCGA-LUAD cohort were downloaded using the R TCGAbiolinks package. A total of 90 clinical couples from the case-control study were collected to analyze the relationship between exosomal miR-149-5p expression and clinical information. All patients were informed of the study and provided written informed consent. The experiment was approved by the Institutional Review Board of China Medical University (No.67 in 2010) and conducted using methodologies conforming to the standards set by the Declaration of Helsinki.

### Construction of WGCNA co-expression network

WGCNA co-expression network was constructed via the R WGCNA package. Exosomal miRNAs were classified into different modules. The soft threshold was calculated when scale-free R^2^>0.9. Then the cluster dendrogram and network heatmap of selected miRNAs were executed. Eigengene values of modules were calculated and the eigengene adjacency heatmap was carried out.

### Pathway prediction and GSEA

DIANA-miRPath provides miRNA pathway analyses and accurate statistics. Pathways of exosomal miRNAs in each module were predicted. The prediction of pathways for AMOTL2 was performed via GSEA software.

### Exosomes isolation and identification

Exosomes were isolated by Minute™ Hi-Efficiency Exosome Precipitation Reagent (Invent EI-027) according to the protocol and then filtered with a 0.22 μm filter to purify the exosomes. The exosomes were identified by transmission electron microscope observation and exosomal protein biomarkers. The exosomes were fixed with 4% glutaraldehyde at 4° C for 2h, and then rinsed three times with 0.1mol/L PBS and fixed with 1% osmium tetroxide for 2h. Next, dehydrated by conventional ethanol and gradient acetone, exosomes were immersed, embedded, polymerized in epoxy resin and then observed under a transmission electron microscope. The proteins from exosomes were lysed with cell lysis buffer (Takara 635656). CD63 (Abcam 125011) and TSG101 (Abcam 134045) were used as exosome markers.

### Cells culture

HBE and A549 cells were cultured in RPMI 1640 media with 10% FBS at 37° C with 5% CO_2_.

### GW4869

A549 cells were divided into two groups: GW4869 group and control group. 1*10^6^ cells were seeded into culture dish and cultured for 24h. Cells were treated with GW4869 for 24h and then cultured in RPMI 1640.

### RNA isolation and RT-PCR

Total RNA was isolated by RNAiso Plus (TAKARA 9108). Reverse transfection was conducted according to the TAKARA 638313 kit manufacturer's instructions. And then RT-PCR was performed using TAKARA RR820A kit. The sequence of miR-149-5p was as follows: Forward 5’-GTCTTCACTCCCGTGCTTGT-3’; Reverse 5’-CCCGAAACACCCGTAAGATA-3’.

### Western blot

Exosomes were lysed in RIPA buffer (Cell Signaling Technology, Danvers, MA). The total proteins were separated by 10% SDS-PAGE and transferred to PVDF membranes. The membranes were incubated with primary antibodies at 4° C overnight with CD63 diluted 1:2000 (Abcam 217345) and TSG101 diluted 1:1000 (Abcam 125011). Then the membranes were incubated with the corresponding HRP-conjugated secondary antibodies for 2 hours at room temperature.

### Cells transfection

A549 cells were divided into three groups: miR-149-5p-mimic, miR-149-5p-inhibitor and NC. Plasmids were designed from Syngeneiech (Beijing, China). siRNA-AMOTL2 was used to knockdown the expression of AMOTL2. 1*10^5^ cells were seeded into 6-well plates and transfected plasmids using jetPRIME transfection reagent (Ployplus transfection). After transfection 24 hours, cells were collected for the following experiments.

### Cells proliferation assays

Cells proliferation was tested using MTS (Promega G3580) according to manufacturer`s instructions. 5*10^3^ cells were seeded into 96-well plates and cultured for 0-72h. 20μL MTS solution was added into each well and incubated for 3h at 37° C with 5% CO_2_. The OD was measured at 490nm.

### Cells apoptosis assays

The cells were harvested when they reached 80% confluence and washed with PBS twice. Annexin V-APC/7-AAD kit (KeyGEN BioTECH KGA1023) was used to test treated cells apoptosis according to manufacturer`s instructions. The percentage of apoptotic cells were assayed by flow cytometry.

### Dual luciferase reporter assay

The AMOTL2 mRNA 3′-UTR WT (5'-UUGGUCAUAAACCCUGAGCCAGA-3') containing the miR-149-5p binding sites (3'-CCCUCACUUCUGUGC CUCGGUCU-5') was subcloned into the pGL3 miReport vector (Genechem, Shanghai, China). The luciferase activity was determined by the Dual-Luciferase Reporter Assay System (Promega, USA).

### Identification of target genes of exosomal miR-149-5p

Targetscan (http://www.targetscan.org/vert_72/), miRWalk (http://mirwalk.umm.uni-heidelberg.de/), miRDB (http://mirdb.org/) and miRDIP (http://ophid.utoronto.ca/mirDIP/index_confirm.jsp) bioinformatic websites were performed to predict the target genes of exosomal miR-149-5p. Kaplan-Meier Plotter was used to assess the overall survival of target genes in lung adenocarcinoma.

### Statistics analysis

Differences between two groups were compared using T-test. Differences among multiple groups were compared using one-way analysis of variance. Univariate and multivariate analyses were performed by SPSS 20.0. A value of p<0.05 was considered to be statistically significant.
