# Correction for: Integrative analysis of exosomal microRNA-149-5p in lung adenocarcinoma

**DOI:** 10.18632/aging.203836

**Published:** 2022-01-15

**Authors:** Wen Tian, He Yang, Baosen Zhou

**Affiliations:** 1 Department of Clinical Epidemiology, First Affiliated Hospital, China Medical University, Shenyang, China; 2 Department of Epidemiology, School of Public Health, China Medical University, Shenyang, China

Original article: Aging. 2021; 13:7382–7396. 7382-7396 . https://doi.org/10.18632/aging.202596

**This article has been corrected:** The authors requested replacement of panel **6B**, in which the left panel (normal tissue stained for BCL2L2) was an accidental duplication of the left panel of **Figure 6A** (normal tissue stained for AMOTL2). All of the results in **Figure 6** were extracted from THPA online database (https://www.proteinatlas.org/). The corrected web link for the left panel of **Figure 6B** is https://www.proteinatlas.org/ENSG00000129473-BCL2L2/tissue/lung#img. This change does not affect the results or conclusions of this work.

New **Figure 6** is presented below.

**Figure 6:**
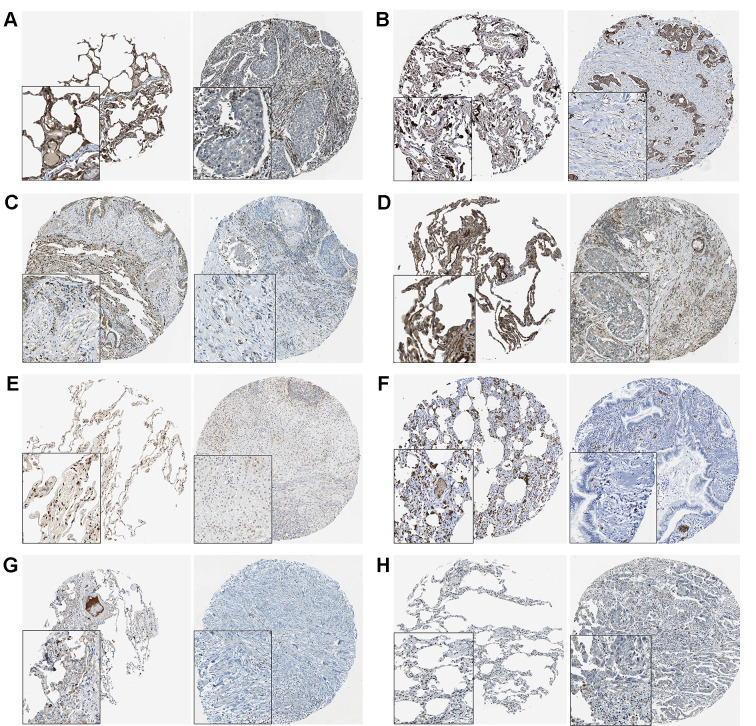
**The THPA results of 8 target genes in normal and tumor tissues.** (**A**) AMOTL2. (**B**) BCL2L2. (**C**) CACDH1. (**D**) MSRB3. (**E**) NFIB. (**F**) S1PR2. (**G**) SORT1. (**H**) SRF.

